# Selection Bias in Real-World Data Studies Used to Support Health Technology Assessments: A Case Study in Metastatic Cancer

**DOI:** 10.3390/curroncol30020151

**Published:** 2023-02-06

**Authors:** Tamer N. Jarada, Dylan E. O’Sullivan, Darren R. Brenner, Winson Y. Cheung, Devon J. Boyne

**Affiliations:** 1 Department of Oncology, University of Calgary, Calgary, AB T2N 4N1, Canada; 2 Oncology Outcomes Initiative, University of Calgary, Calgary, AB T2N 4N1, Canada; 3 Department of Community Health Sciences, University of Calgary, Calgary, AB T2N 4N1, Canada

**Keywords:** real-world evidence, selection bias, oncology, survival, treatment patterns

## Abstract

Real-world evidence has been increasingly used to support evaluations of emerging therapies. These investigations are often conducted in settings that may not be representative of the underlying population. The purpose of this investigation was to empirically quantify the magnitude of this selection bias. Individuals diagnosed with solid metastatic cancer in Alberta, Canada, between 2010–2019 were identified using the provincial cancer registry for 13 common metastatic sites. Two outcomes used to support oncology reimbursement decisions were examined: the proportion of individuals who initiated systemic therapy and median overall survival (OS). These outcomes were assessed in the entire population and in a subset of individuals who were referred to a medical oncologist. Among the 23,152 individuals in the entire population, 40.8% (95% CI: 40.2–41.4) initiated systemic therapy, and the median OS from diagnosis was 5.4 months (95% CI: 5.3–5.6). Among those who were referred to a medical oncologist (*n* = 13,372; 57.8%), 67.4% (95% CI: 66.6–68.2) initiated systemic therapy, and the median OS from diagnosis was 11.2 months (95% CI: 10.9–11.5). The magnitude of bias varied by cancer site where lower referral rates were associated with greater bias. Non-referral is an important source of selection bias in real-world investigations. Studies that rely on limited-catchment real-world data should be interpreted with caution, particularly in metastatic cancer settings.

## 1. Introduction

With the advent of electronic medical records (EMR), billing claims databases, and their linkage with disease registries, real-world evidence (RWE) is increasingly being used to inform medical, regulatory, and reimbursement decision making [1,2,3,4,5,6,7,8]. Examples include the leveraging of RWE pertaining to treatment patterns and outcomes to help establish an unmet clinical need for alternative treatment options and to inform economic modeling or to help reassess the continued funding of or expanded access to new therapies after they have been introduced into clinical practice [1,2,3,4,7,8].

A variety of data sources can be used to generate RWE [5]. We define population-based real-world data (PbRWD) as data that provides complete coverage of all individuals with a condition of interest within a specified geographic area in a given timeframe. Examples of such data sources include disease registries in regions with mandatory reporting or administrative-claims data in single-payer healthcare settings. In many regions and disease settings, PbRWD are not available, and researchers must instead rely on limited-catchment real-world data (LcRWD) that captures data on a selected subset of individuals from the underlying study population. Examples of LcRWD include the use of electronic medical records or chart review data from select treatment centres that do not provide complete coverage for a given study population or the use of physician surveys and medical advisory boards to collect information about patients seen within the respondents’ clinical practice.

In comparison to PbRWD, RWE generated using LcRWD may have a higher risk of selection bias and lack of generalizability [9,10,11]. For example, treatment patterns in academic or southern treatment centres may not reflect that of their community or northern counterparts. Similarly, the outcomes of individuals referred to a specialist for treatment may be more favorable than that of the underlying population due to the systematic exclusion of individuals who were not referred for treatment because their disease was too advanced, they were too frail, or they refuse treatment. As a result, studies that attempt to characterize treatment patterns and outcomes may not be generalizable if they rely on data from LcRWD.

Given the increasing role of RWE to support health technology assessment, it is important to understand the risk of selection bias associated with the use LcRWD. The purpose of this investigation was to empirically quantify the magnitude of this selection bias in an applied oncology setting for two outcomes: (1) the proportion of patients who initiate systemic therapy; and (2) the median overall survival from diagnosis. We estimated these outcomes using PbRWD and compared the findings with an emulated LcRWD scenario in which we restricted the analyses to patients who were referred to a medical oncologist.

We speculated that the two outcomes would be systematically overestimated when estimated using data from medical oncology clinics. Specifically, we hypothesized that the denominator used to estimate the proportion of individuals who initiate therapy would be too small in the LcRWD due to the exclusion of individuals who were never referred for treatment, resulting in an inflated estimate. Similarly, we hypothesized that estimates of survival from the LcRWD would be artificially high due to the non-referral of individuals with worse prognoses and the exclusion of individuals who died before having the opportunity to see a medical oncologist.

Our main motivation for conducting this investigation was to systematically address skepticism towards results from PbRWD analyses by highlighting the potential magnitude of selection bias with LcRWD in a common oncology setting. We chose to focus on selection bias resulting from non-referral to a medical oncologist because this is a potentially common type of bias that may arise in metastatic cancer RWD settings, whereby a large proportion of individuals are systematically excluded from the analyses because they were never referred for treatment. This selection bias may lead to distorted perspectives amongst clinicians who treat cancer, regarding the proportion who initiate treatment and survival outcomes at the population-level. Findings from this investigation will help to contextualize RWE from single- or multi-centre studies, whereby the estimated rate of treatment and median overall survival may be artificially high when compared the true population-level estimates due to non-referral.

## 2. Materials and Methods

A retrospective cohort study was conducted using PbRWD from Alberta, Canada. All adults aged ≥18 years who were newly diagnosed with a metastatic solid tumor within Alberta, Canada, between 2010 to 2019 were identified using the provincial cancer registry. Certified by the North American Association of Central Cancer Registries, the provincial cancer registry captures data for all diagnoses of cancer within the province because cancer is a reportable event in Alberta and includes accurate information on the disease stage and time of diagnosis. The 13 most common solid metastatic tumor types examined were bladder, breast, colorectal, endometrium, esophagus, kidney, liver, lung, melanoma of the skin, ovary, pancreas, prostate, and stomach. Registry data were linked to provincial electronic medical records to identify receipt of systemic therapy and referral to a medical oncologist any time after initial diagnosis. Data linkage was accomplished using unique lifetime identifier numbers which are assigned to all Alberta residents. The provincial electronic medical records provide coverage for all 17 cancer centres within the province which comprise two tertiary cancer centres, 11 community cancer centres, and four regional cancer centres. Individuals were followed from the time of initial diagnosis until death from any cause (ascertained via vital statistics), last known contact with the healthcare system, or 31 December 2020, whichever came first. Individuals missing data on the disease stage were excluded. In this study, the PbRWD cohort included the full study population, while the LcRWD cohort only included patients that were referred to a medical oncologist any time after their initial cancer diagnosis.

The proportion of patients who received systemic therapy after diagnosis and the median overall survival from diagnosis in the PbRWD and LcRWD cohorts were estimated and compared, both overall and by cancer site. Median overall survival from the time of initial diagnosis until death from any cause was estimated using the Kaplan–Meier method. The 95% confidence intervals were estimated using the exact method for proportions and the log method for median overall survival. The absolute difference and the relative ratio of the estimates of the proportion of individuals who initiate treatment and the median overall survival between the LcRWD and PbRWD were used to quantify the magnitude of bias. Given their direct relation, the magnitude of relative bias was modeled as a function of the referral rate across cancer sites. This exploratory analysis was conducted using weighted linear regression, whereby the weights were defined by the number of individuals in each cancer site.

## 3. Results

Between January 2010 and December 2019, 23,152 cases of metastatic cancer were diagnosed for the top 13 most common metastatic solid tumors in Alberta. The most common metastatic solid tumors were lung (43.1%), colorectum (16.6%), and pancreas (9.4%), which collectively accounted for 69.1% of cancers included in this study. Across all tumor groups, 57.8% of patients were referred to a medical oncologist (*n* = 13,372), ranging from 43.5% for liver cancer to 84.3% for endometrial cancer (Table 1).

Estimates of treatment initiation were greatly inflated in the LcRWD compared to that of the underlying population-based study population (PbRWD). In the PbRWD, 40.8% (95% CI: 40.2–41.4) of patients initiated systemic therapy, ranging from 18.4% (95% CI: 14.1–23.5) for liver cancer to 76.5% (95% CI: 74.1–788) for breast cancer (Table 1). In contrast, the estimated rate of systemic therapy use in the LcRWD was 67.4% (95% CI: 66.6–68.2), ranging from 42.2% (95% CI: 33.3–51.5) for liver cancer to 91.3% (95% CI: 89.4–92.9) for breast cancer. Treatment use in the LcRWD was 1.65 (95% CI: 1.64–1.67) times greater than that of the PbRWD, ranging from 1.15 (95% CI: 1.10–1.20) for endometrial cancer to 2.30 (95% CI: 2.02–2.65) for liver cancer, and the absolute difference in treatment use estimates in the LcRWD and the PbRWD was 26.63% (95% CI: 26.08–27.17), ranging from 9.87% (95% CI: 6.76–13.10) for endometrial cancer to 30.72% (29.79–31.67) for colorectal cancer.

Similar to treatment with systemic therapy, the median overall survival from diagnosis in the LcRWD cohort was considerably greater than the underlying population-based study population. Median overall survival from diagnosis in the PbRWD cohort was 5.42 months (95% CI: 5.29–5.59), ranging from 2.27 months (95% CI: 2.10–2.47) for pancreatic cancer to 26.86 months (95% CI: 25.08–28.50) for prostate cancer, whereas the median overall survival in the LcRWD cohort was 11.24 months (95% CI: 10.88–11.54), ranging from 4.04 months (95% CI: 3.68–4.44) for pancreatic cancer to 33.47 months (31.73–36.03) for prostate cancer (Table 2). Median overall survival in the LcRWD was 2.07 (95% CI: 2.00–2.13) times greater than that of the PbRWD, ranging from 1.24 (95% CI: 1.18–1.37) for breast cancer to 2.60 (95% CI: 1.89–3.63) for liver cancer, and the absolute difference in median overall survival in the LcRWD and the PbRWD was 5.82 (95% CI: 5.49–6.08) months, ranging from 1.78 (95% CI:1.45–2.14) months for pancreatic cancer to 8.22 (95% CI: 5.52–10.68) months for kidney cancer.

**Figure 1:**
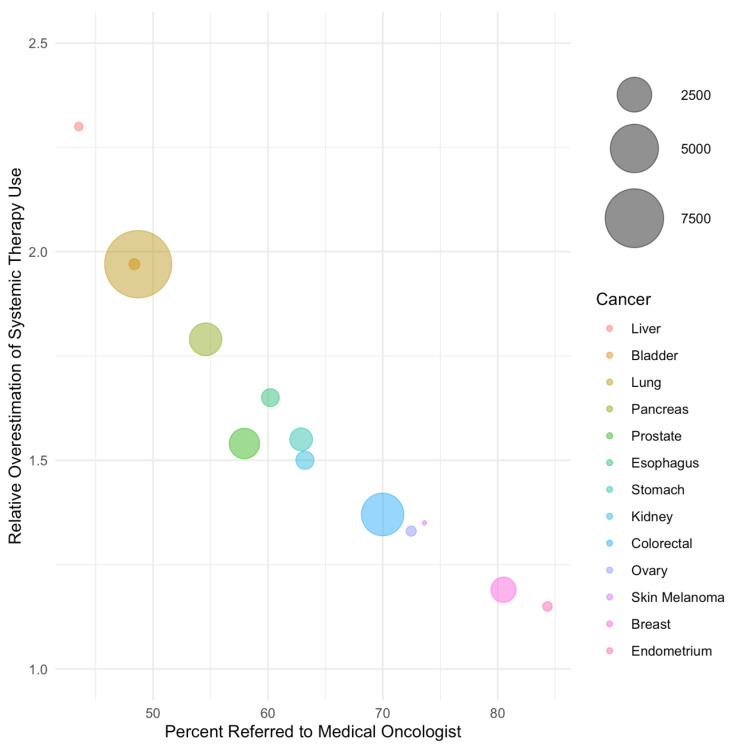
The relative overestimation of systemic treatment uptake in samples of referred patients compared to population-based samples by cancer site. Cancer sites are listed in order of ascending referral rate and bubble sizes are proportional to the number of patients in each cancer site.

**Figure 2:**
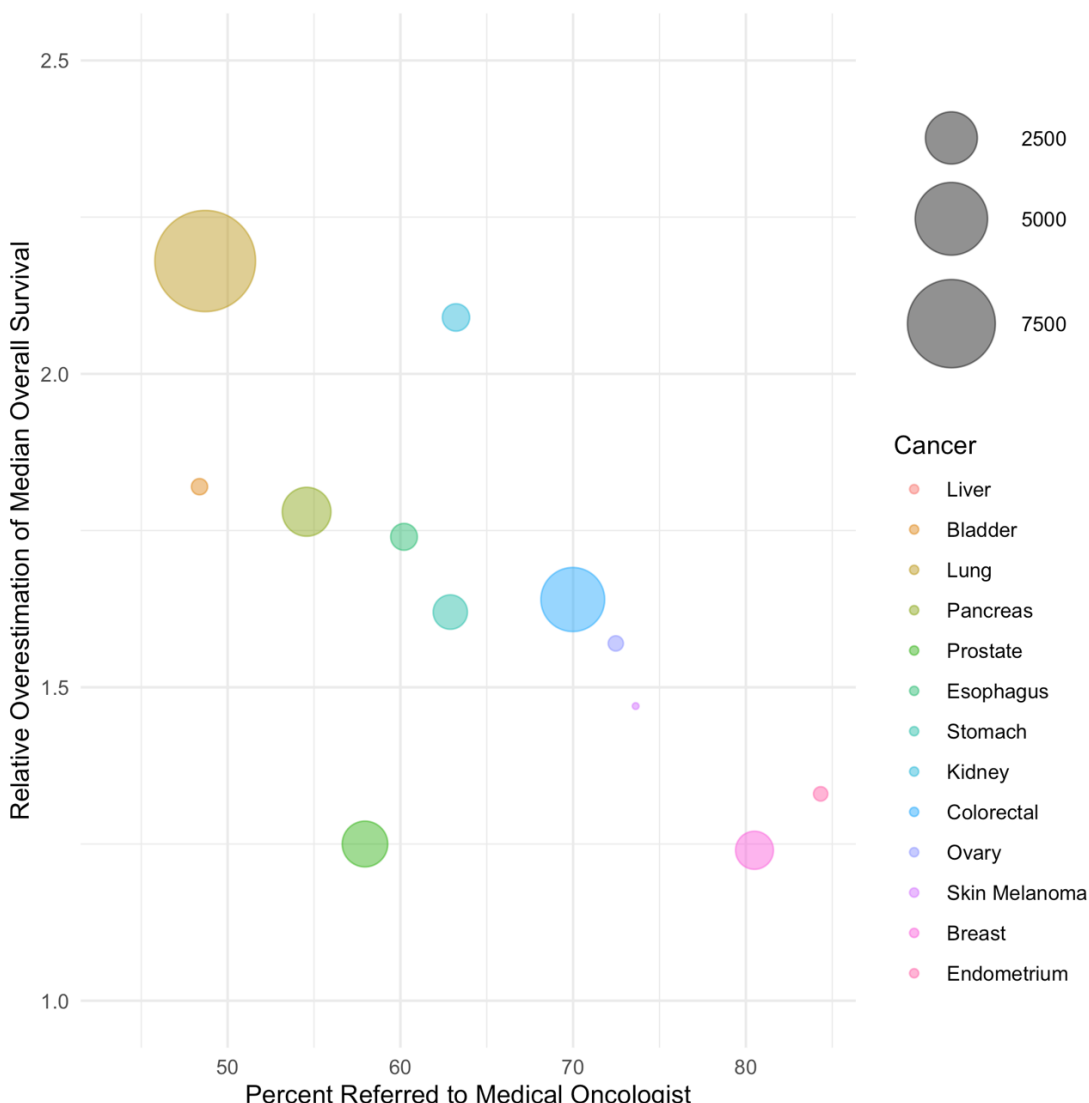
The relative overestimation of median overall survival in samples of referred patients compared to population-based samples by cancer site. Cancer sites are listed in order of ascending referral rate and bubble sizes are proportional to the number of patients in each cancer site.

In weighted regression analysis, the rate of referral was linearly associated with the magnitude of bias (Figure 1 and Figure 2, Table 3). A 50% referral rate was found to correspond to estimates of systemic therapy initiation and median overall survival that were 1.91 (95% CI: 1.86–1.97) and 2.06 (95% CI: 1.88–2.24) times greater in the LcRWD than in the PbRWD, respectively (Table 3). In contrast, an 80% referral rate was found to correspond to estimates of systemic therapy initiation and median overall survival that were 1.12 (95% CI: 1.02–1.22) and 1.27 (95% CI: 0.94–1.60) times greater in the LcRWD than in the PbRWD.

## 4. Discussion

In this investigation, the magnitude of selection bias associated with the use of LcRWD in a common metastatic cancer setting was empirically quantified for two metrics often used to support health technology assessment submissions. The degree of bias was considerable and varied by cancer site. The degree of overestimation was directly linked to the rate of non-referral which tended to be higher for more fatal cancers such as pancreatic, liver, and lung cancer. For these three sites, the estimated proportion of individuals who initiated systemic therapy and the median OS in the LcRWD was approximately double that of the PbRWD. Even for less fatal cancers with higher rates of referral, the absolute difference in treatment initiation and median OS was often greater than 10–20% which could still meaningfully impact downstream pharmacoeconomic modeling and decision making.

These findings underscore the importance of using PbRWD when generating descriptive RWE related to patient characteristics, treatment patterns, and outcomes. Since this investigation was conducted in a universal healthcare setting, we suspect that the degree of non-referral and the worse prognosis of individuals who were not referred to a medical oncologist may be more pronounced in regions that do not provide coverage for cancer treatment. Descriptive studies that rely on LcRWD should therefore be interpreted with caution, particularly in situations where the sampling process is not well understood and has not been accounted for in the analysis (e.g., via sampling weights).

In situations where PbRWD is unavailable, results from this investigation may help to inform on the potential magnitude of bias associated with the reliance on descriptive LcRWD in metastatic cancer settings, particularly those that rely on data captured from a single treatment centre or practice. In our investigation, the magnitude of overestimation when relying on data from patients who were referred for consultation with a medical oncologist was 1.7 times greater (range: 1.2 to 2.3) when estimating the proportion of individuals who initiate treatment and 2.1 times greater (range: 1.2 to 2.6) when estimating the median overall survival from diagnosis relative to the true population-level estimates. These bias parameters may help to contextualize future studies that have relied upon LcRWD by providing a range of plausible values for the relative degree of overestimation of the true population-level estimates. For example, if a single treatment centre study estimated that 90% of metastatic small-cell lung cancer cases referred to their clinic received systemic therapy, findings from our investigation suggests that the true population-level estimate would be closer to 46% (i.e., 90%/1.97 [bias parameter for lung cancer]).

Our study has limitations. First, the estimated magnitude of bias reported in this investigation may not be generalizable to other research settings. Additional research expanding these results to other types of illnesses, outcomes, and other LcRWD scenarios can help to further our understanding of the potential risk of bias associated with the reliance on LcRWD. Second, the focus of our investigation was on a descriptive assessment of an entire population of cancer patients. These findings may not be applicable to studies focused on estimating the comparative efficacy of different treatment strategies or focused on outcomes among individuals who initiated a specific type of treatment. Third, we lacked information on referral to urologists who practice outside of the cancer system and who sometimes administer systemic therapy for bladder, kidney, and prostate cancer. Our analysis likely underestimated the proportion of individuals referred for systemic treatment for these disease sites, which limits the generalizability of our emulated LcRWD scenario to other settings in which urologists are included in the analyses. Lastly, the cancer registry only captures information at the time of initial diagnosis, and our investigation did not include individuals who presented with an early-stage disease but included those whose diseases later progressed or had a recurrence. These results are therefore specific to newly diagnosed cancer patients and may not be generalizable to individuals with a recurrent disease.

## 5. Conclusions

Non-referral is an important source of bias in descriptive real-world studies of cancer treatment patterns and outcomes. This bias is particularly of concern among studies of metastatic cancers focused solely on individuals referred to a medical oncologist. Systemic therapy initiation and overall survival from diagnosis among individuals with metastatic cancer were considerably overestimated when restricted to patients who were referred to a medical oncologist, and the bias was highest among cancer sites with the lowest referral rates. Descriptive real-world evidence generated from limited catchment databases should be interpreted with caution.

## Figures and Tables

**Table 1:** Difference in the proportion of newly metastatic-diagnosed cancer patients in Alberta treated with systemic therapy (2010–2019) at the population-level versus among individuals referred to medical oncologists.

Tumor Site	Population-Based Real-World Data (PbRWD)		Limited Catchment Real-World Data (LcRWD)		Bias from a Sample of Referred Patients	
	No. of Patients	Initiated Therapy, *N* (%)	No. of Patients ^1^ (%)	Initiated Therapy, *N* (%)	Relative Bias	Absolute Bias
All Sites	23,152	9444 (40.79)	13,372 (57.76)	9015 (67.42)	1.65	26.63
Lung	9985	3170 (31.75)	4863 (48.70)	3038 (62.47)	1.97	30.72
Colorectal	3831	1989 (51.92)	2681 (69.98)	1912 (71.32)	1.37	19.40
Pancreas	2186	567 (25.94)	1193 (54.57)	555 (46.52)	1.79	20.58
Prostate	1905	1085 (56.96)	1104 (57.95)	966 (87.50)	1.54	30.54
Breast	1313	1005 (76.54)	1057 (80.50)	965 (91.30)	1.19	14.76
Stomach	1078	362 (33.58)	678 (62.89)	354 (52.21)	1.55	18.63
Kidney	707	367 (51.91)	447 (63.22)	348 (77.85)	1.50	25.94
Esophagus	681	220 (32.31)	410 (60.21)	218 (53.17)	1.65	20.86
Bladder	337	108 (32.05)	163 (48.37)	103 (63.19)	1.97	31.14
Ovary	316	192 (60.76)	229 (72.47)	185 (80.79)	1.33	20.03
Endometrium	300	204 (68.00)	253 (84.33)	197 (77.87)	1.15	9.87
Liver	278	51 (18.35)	121 (43.53)	51 (42.15)	2.30	23.80
Skin Melanoma	235	124 (52.77)	173 (73.62)	123 (71.10)	1.35	18.33

^1^ Number of patients referred to a medical oncologist.

**Table 2:** Difference in the median overall survival from diagnosis of newly metastatic-diagnosed cancer patients in Alberta treated (2010–2019) at the population-level versus among individuals referred to medical oncologists.

Tumor Site	Population-Based Real-World Data (PbRWD)		Limited Catchment Real-World Data (LcRWD)	Bias from a Sample of Referred Patients		
	No. of Patients	Median OS (95% CI)	No. of Patients ^1^	Median OS (95% CI)	Relative Bias	Absolute Bias
All Sites	23,152	5.42 (5.29 to 5.59)	13,372 (57.76)	11.24 (10.88 to 11.54)	2.07	5.82
Lung	9985	3.78 (3.62 to 3.95)	4863 (48.70)	8.25 (7.99 to 8.58)	2.18	4.47
Colorectal	3831	10.32 (9.76 to 11.01)	2681 (69.98)	16.90 (16.01 to 17.98)	1.64	6.58
Pancreas	2186	2.27 (2.10 to 2.47)	1193 (54.57)	4.04 (3.68 to 4.44)	1.78	1.78
Prostate	1905	26.86 (25.08 to 28.50)	1104 (57.95)	33.47 (31.73 to 36.03)	1.25	6.61
Breast	1313	26.10 (24.30 to 28.41)	1057 (80.50)	32.35 (30.51 to 36.72)	1.24	6.25
Stomach	1078	3.98 (3.62 to 4.54)	678 (62.89)	6.44 (5.98 to 7.04)	1.62	2.47
Kidney	707	7.53 (6.08 to 9.24)	447 (63.22)	15.75 (13.15 to 18.48)	2.09	8.22
Esophagus	681	4.11 (3.65 to 4.67)	410 (60.21)	7.13 (6.28 to 7.76)	1.74	3.02
Bladder	337	4.50 (3.68 to 5.10)	163 (48.37)	8.19 (6.58 to 10.95)	1.82	3.68
Ovary	316	11.41 (7.50 to 14.89)	229 (72.47)	17.88 (16.27 to 20.22)	1.57	6.48
Endometrium	300	10.32 (8.61 to 13.22)	253 (84.33)	13.71 (10.55 to 17.03)	1.33	3.39
Liver	278	2.47 (1.87 to 3.35)	121 (43.53)	6.41 (4.96 to 8.12)	2.60	3.95
Skin Melanoma	235	8.55 (6.38 to 11.24)	173 (73.62)	12.53 (10.42 to 18.71)	1.47	3.98

^1^ Number of patients referred to a medical oncologist.

**Table 3:** Results from weighted regression analysis estimating the magnitude of relative bias for treatment initiation and median overall survival associated with different rates of referral to a medical oncologist in Alberta, Canada.

Percentage of Individuals Refferred to a Medical Oncologist	Relative Bias in Percent of Individuals Who Initiated Systemic Therapy (95% CI)	Relative Bias in Median Overall Survival (95% CI)
50%	1.91 (95% CI: 1.86 to 1.97)	2.06 (95% CI: 1.88 to 2.24)
60%	1.65 (95% CI: 1.61 to 1.69)	1.80 (95% CI: 1.65 to 1.94)
70%	1.39 (95% CI: 1.32 to 1.45)	1.53 (95% CI: 1.32 to 1.75)
80%	1.12 (95% CI: 1.02 to 1.22)	1.27 (95% CI: 0.94 to 1.60)

## Data Availability

Individual-level data are not publicly available due to Canadian data privacy laws governing personal health information.
